# Editorial: Exploring the developmental plasticity and transgenerational effects on the thermal biology of aquatic ectotherms

**DOI:** 10.3389/fphys.2024.1507027

**Published:** 2024-10-16

**Authors:** Yangfan Zhang, Katja Anttila, Anthony J. Hickey

**Affiliations:** ^1^ Museum of Comparative Zoology, Department of Organismic and Evolutionary Biology, Harvard University, Cambridge, MA, United States; ^2^ Department of Biology, University of Turku, Turku, Finland; ^3^ School of Biological Sciences, The University of Auckland/Waipapa Tamata Rau, The University of Auckland, Auckland, New Zealand

**Keywords:** phenotypic plasticity, aquatic, global climate change, ectotherm, microbial

As the projected 1.5°C warmer world is likely to be realized, scientific studies exploring response strategies of animals to warmer climates are shifting from mechanisms of phenotypic plasticity, to those of adaptation at the genomic level. Physiological adjustments that shift with environmental temperature increases may also persist given the impacts on developmental trajectories plasticity and transgenerational effects such as through nutritional deficits placed on gametes and genomic imprinting. This short Research Topic includes four model organisms: purple sea urchins (*Strongylocentrotus purpuratus*), Olympia oysters (*Ostrea lurida*), green-lipped mussels (*Perna canaliculus*) and sharks to gauge an understanding of developmental plasticity and transgenerational potential of thermal biology from aquatic invertebrates to elasmobranch fishes.

Purple sea urchins in the kelp forests of the California Current System have experienced marine heat waves that overlap with their developmental stages. This scenario provides a model system to study the transgenerational plasticity of thermal tolerance in invertebrates. The study showed that embryos from females experiencing prolonged marine heatwave events increase thermal tolerance relative to those from females not experiencing prolonged marine heatwaves. More thermally tolerant embryos resulted from eggs with higher protein concentrations (Chamorro et al.). This study revealed a potential for transgenerational adaptation to heat waves in sea urchins through maternal effects on the protein content of the eggs.

Green-lipped mussels in New Zealand are also experiencing increased frequencies of marine heat waves and extreme weather events. Here, Ericson et al. show that such events correlate with increased stress-related mortality. They also reveal that the microbiome is an important factor that is associated with green-lipped mussel survival. The family of mussels with the highest survival in the face of thermal stress differed from others in terms of microbiome species-richness, despite gene expression profiles for heat shock proteins and immune responses being similarly upregulated in all families (Ericson et al.). This study highlights the importance of considering the roles of the microbiome when monitoring the impacts of thermal stress on ectotherms.

Alma et al. revealed that spatiotemporal variation in the ocean environmental conditions affects the phenotype of the adult Olympia oysters, and identified carry-over parental environmental history effects on their offspring. Authors out-planted one-year-old oysters in three locations in Washington State that differed in the temporal variation of temperature, oxygen, salinity, chlorophyll, and carbon chemistry. They measured oyster growth, lipid content, reproduction status, and standard metabolic rate (SMR, metabolic measurements conducted at five different temperatures and pH levels) 6 & 12 months after out-planting. Survival and growth of their larvae were measured at 14°C and 20°C, and the SMR at five different temperatures. The temporal variation of the environmental conditions impacted adult oyster growth and reproduction, and metabolic plasticity in response to acute temperature and pH changes. Importantly, larval survival, growth, and SMR at different temperatures are associated with parental sites, which highlights the influence of ocean conditions on the fitness of oysters and that effects may persist over the generations.

Hasenei et al. contend that the phenotypic plasticity in the face of warming waters may play a key role in the survival of elasmobranchs. Because of their long life spans, late sexual maturity and low fecundity, our understanding of transgenerational plasticity within elasmobranchs is poor. The perspective of Hasenei et al. highlights a selection of research areas and candidate new technologies that aim to fill in knowledge gaps.

As highlighted by this Research Topic, aquatic invertebrate models are at the frontline of the research on developmental plasticity and transgenerational effects on the thermal biology of aquatic ectotherms. While comparatively short generational times present promising and model organisms that facilitate the study of transgenerational effects, elasmobranchs showcase challenges in studying the transgenerational plasticity species with contrasting long life spans, late sexual maturation and low fecundity. These organisms demonstrated that aquatic ectotherms can manifest developmental plasticity, which shapes the phenotype within a generation. Then they illustrate transgenerational carryover effects that impact phenotype. At least in the purple urchin, a predominated component influencing transgenerational effects correlates with egg protein compositions. In addition, microbial diversity adds another layer of complexity to green-lipped mussels. This indicates that symbiotic relationships warrant consideration as drivers of developmental plasticity and transgenerational effects. A perspective paper summarizes the case studies and some future directions for tackling the challenging research area of transgenic aspects of thermal biology on elasmobranchs. While we are distant from predicting whether the new steady state of a phenotypic trait is heritable or is simply a transient plastic response, this Research Topic hopefully inspires future studies to disentangle the transgenerational genomic changes and the epigenetic effects.

## Generative AI statement

The author(s) declare that no Generative AI was used in the creation of this manuscript.

